# Easy to Use Plastic Optical Fiber-Based Biosensor for Detection of Butanal

**DOI:** 10.1371/journal.pone.0116770

**Published:** 2015-03-19

**Authors:** Nunzio Cennamo, Stefano Di Giovanni, Antonio Varriale, Maria Staiano, Fabio Di Pietrantonio, Andrea Notargiacomo, Luigi Zeni, Sabato D’Auria

**Affiliations:** 1 Department of Industrial and Information Engineering, SUN, Aversa, Italy; 2 Institute of Protein Biochemistry, CNR, Napoli, Italy; 3 “O.M.Corbino“ Institute of Acoustics and Sensors, Rome, Italy; 4 Institute for Photonics and Nanotechnologies, CNR, Rome, Italy; 5 Institute of Food Science, CNR, Avellino, Italy; University of Oldenburg, GERMANY

## Abstract

The final goal of this work is to achieve a selective detection of butanal by the realization of a simple, small-size and low cost experimental approach. To this end, a porcine odorant-binding protein was used in connection with surface plasmon resonance transduction in a plastic optical fiber tool for the selective detection of butanal by a competitive assay. This allows to reduce the cost and the size of the sensing device and it offers the possibility to design a “Lab-on-a-chip” platform. The obtained results showed that this system approach is able to selectively detect the presence of butanal in the concentration range from 20 μM to 1000 μM.

## Introduction

Aldehydes are some of the main toxic compounds present in the environment and together with other compounds represent the water-soluble fraction of the Urban Particulate Matter (PM). In fact low-molecular mass aldehydes are widespread in air, water, alcohol and industrial waste material as consequence of the human activity [1]. These compounds are relatively volatile, polar, reactive and exhibited adverse health effects (carcinogenicity, cytotoxicity) on both mammalian cells and microorganisms. Different studies have demonstrated various aldehydes as irritants upon inhalation, skin contact and are allergens [2]. In the air the characterization of aldehydes in PM could be essential in order to elucidate the complex set of reactions that occur in the atmosphere. On the contrary, the determination of the low-molecular mass aldehydes present in environmental liquids such as wastewater, in relation to their toxic and carcinogenic effect on the human beings, is becoming a very crucial task. Actually the most commonly used methodologies for the determination of aldehydes in PM is the Gas chromatography (GC). Chemical derivatizations are normally employed in order to increase the sensitivity. In particular, one of the methods for aldehyde determination, recommended by the US Environmental Protection Agency (EPA), involves derivatization with *o*-(2,3,4,5,6- pentafluorobenzyl) hydroxylamine hydrochloride (PFBHA) [3,4] after derivatization step of extraction and concentration of the derivate before the GC analysis [1]. Unfortunately, this method is time-consuming, requires complex sample preparation and also uses expensive equipment.

It is very important to develop a low-cost, simple, sensible and accurate method for detection aldehydes in liquid. For this purpose we developed an optical biosensor based on Surface Plasmon Resonance (SPR) in a Plastic Optical Fiber (POF) to detect aldehydes. We used butyraldehyde as a model compound. Among the aldehyde family, butyraldehyde (known also as butanal) is a colourless and acrid smell aliphatic saturated aldehyde. It has been demonstrated that human beings could be exposed to butyraldehyde via inhalation of ambient air, ingestion of food and drinking water, and dermal contact with consumer products containing this compound.

The most popular SPR biosensor systems are based on a high refractive index prism coated with a thin metallic layer (Kretschmann configuration). The incidence angle of the light for which the plasmon resonance is induced depends on the refractive index of the dielectric medium. It can be changed over a wide range, and consequently the excitation of the surface plasma waves (plasmons) may occur whatever the surrounding medium, i.e., a gas or a liquid [5]. These sensors are usually bulky, and not easy to miniaturize and require expensive optical equipments. In principle, these aims can be successfully achieved by using waveguide coupling. An optical fiber makes possible the reduction of the sensor cost and dimensions, with the possibility to integrate the SPR sensing platform with miniaturized optoelectronic devices, eventually leading to a “lab-on-a-chip”.

Actually, several configurations based on SPR in silica optical fibers for measuring the refractive index of aqueous media have been described in the recent literature [6,7]. Furthermore, investigations have also been devoted to Plastic Optical Fibers (POF) as they represent an easier to handle platform, with mechanical properties making them more resilient, cheaper and safer for in vivo determinations.

We recently developed a new geometry for a POF sensor system [8, 9] suitable for bio-applications [10]. POFs are especially advantageous due to their excellent flexibility, easy manipulation, great numerical aperture, large diameter, and the fact that plastic is able to withstand smaller bend radii than glass. The above peculiarities of POFs, that have increased their popularity and competitiveness for telecommunications, are exactly those that are relevant for optical fiber based sensors.

In this work we describe a new SPR-POF-based biosensor for detection of traces of butyraldehyde in solution. For this purpose the gold surface of the chip was chemically modified through the formation of self-assembling monolayer (SAM) using the α-lipoic acid, derivatized with ethylenediamine as spacer group, and finally the butyric acid was covalently bound on the surface of the chip through well known carbodiimide method (EDC/NHS). The obtained results showed that the POF-sensor is able to sense butanal at the concentration less than 25μM.

A competitive assay based on the use porcine Odorant-Binding Protein (pOBP) as molecular recognition element (MRE) able to bind with high affinity the analytes, have been performed in order to detect butanal in solution directly. The Odorant-binding proteins (OBPs) are small extracellular proteins belonging to the lipocalin superfamily able to bind reversibly odorant molecules, which commonly are volatile, small and hydrophobic compounds with no fixed structure and chemical properties [11–14]. Different works have demonstrated their direct implication in the primary step of olfaction function in vertebrate. The members of this protein family have been demonstrated to have a very high stability and are good candidates as molecular recognition elements in protein based biosensor [15–17].

## Results and Discussion

**Fig 1:**
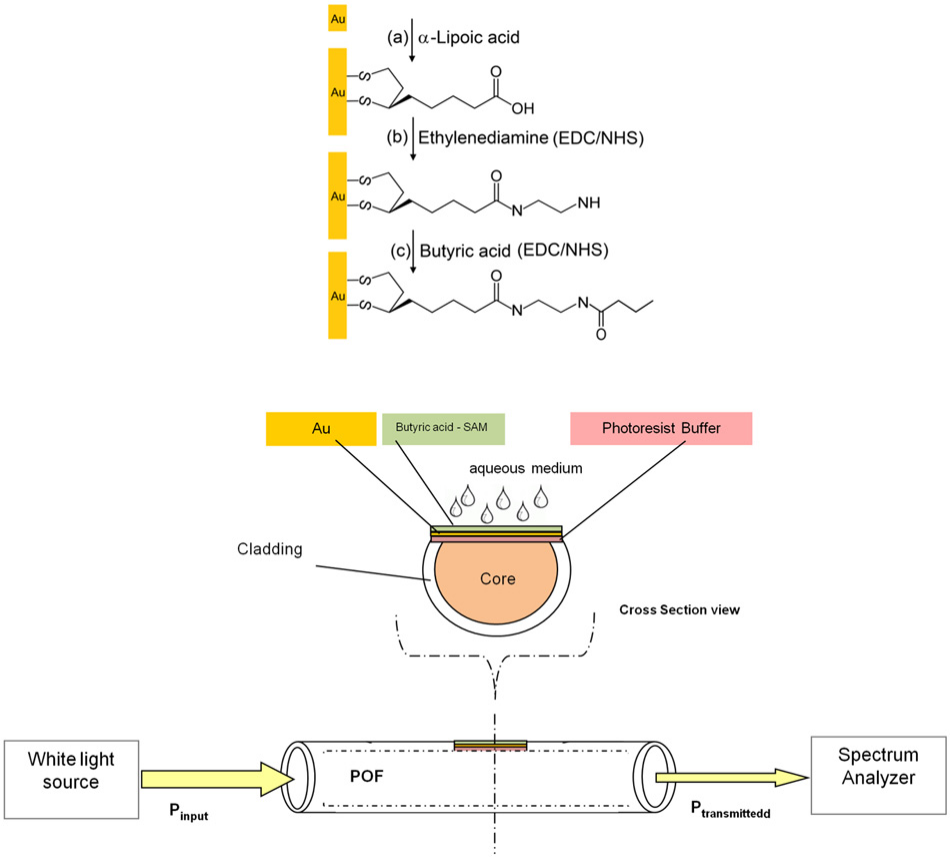
Optical biosensor based on SPR in a POF and experimental setup. Scheme of synthesis of derivatized SAM on chip POF. The gold surface was treated sequentially with a solution of α-lipoic acid (a), ethylenediamine (b) and butyric acid (c).

Fig. 1 shows the SPR-POF platform and the strategy adopted for derivatization of the gold surface chip of the SPR-POF device. In particular, with the aim to develop a competitive assay for butanal, the gold surface was treated sequentially with a solution of α-lipoic acid (a), ethylenediamine (b) and finally with butyric acid (c). The butyric acid was selected for two reasons: it has a structure similar to the butanal, and additionally exhibits a carboxylic group that allows the easy anchoring to the surface. The single steps of surface derivatization were characterized by AFM (Atomic Force Microscopy) measurement.

### 1. AFM Characterization

**Fig 2:**
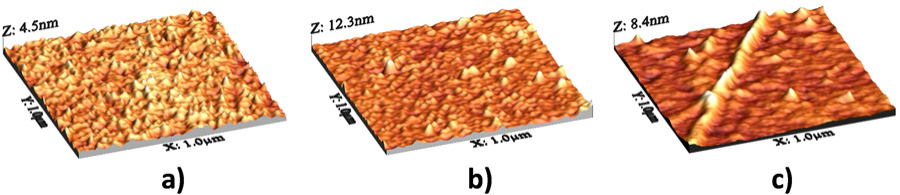
Atomic force microscopy. 3D rendering of: a gold coated Si substrate a) before and b) after functionalization with lipoic acid, and c) after additional subsequent functionalization with butyric acid and pOBP. Note that a different Z scale was used for the three images.

AFM measurments of the sample topography were performed after each fabrication step. The gold coated Si substrate (Fig. 2a) has a grainy like morphology uniformly covering the surface, showing an Rq (roguhness) value of ~0.85nm. After functionalization with lipoic acid (Fig. 2b) rounded clusters appear with diameter of about 50nm and height of ~8–10nm. The Rq surface roughness at this stage has slightly increased to a value of ~1.2nm. On large area scans, random islands or terraces are also present with size of few hundreds of nanometers. Finally, after additional functionalization with butirric acid and pOBP the morphology exhibits a significant change showing a large amount of aggregates. In particular, two kinds of structures protruding from a flat surface are visible: several microns long filaments (Fig. 2c) with diameter of the order of 50nm, and flat aggregates with micrometric size.

### 2. pOBP purification and 1-AMA assay

The gene of the pOBP was cloned, express and the protein was purified. The pOBP presents a folding pattern, an 8-stranded β-barrel flanked by a α-helix at the C-terminal end of the polypeptide chain typical of this class of proteins. The β-barrel creates a central a-polar cavity whose role is to bind and transport hydrophobic odorant molecules. These proteins reversibly bind odorants with dissociation constants in the micromolar range. The purity of different fractions obtained from GST-affinity chromatography step were analyzed by SDS-PAGE (data not shown), the homogeneous fractions were pooled and the molar concentration was determined by absorbance value at λ = 278 nm. The functionalities of the recombinant pOBP were determined by direct titrations using the fluorescent ligand 1-amino-anthracene (AMA). This is the well-used functional assay based on the resonance energy transfer phenomenon between the indolic residues of the protein and the AMA localized in the protein-binding site. In the assay, the indolic residues of pOBP (TRP 16) are excited upon irradiation at 295 nm. Since in the very proximity of them AMA molecules (Forster distance) are located, there is no emission from the indolic residues of OBP, but there is energy transfer to AMA molecules that emit at about 480 nm.

**Fig 3:**
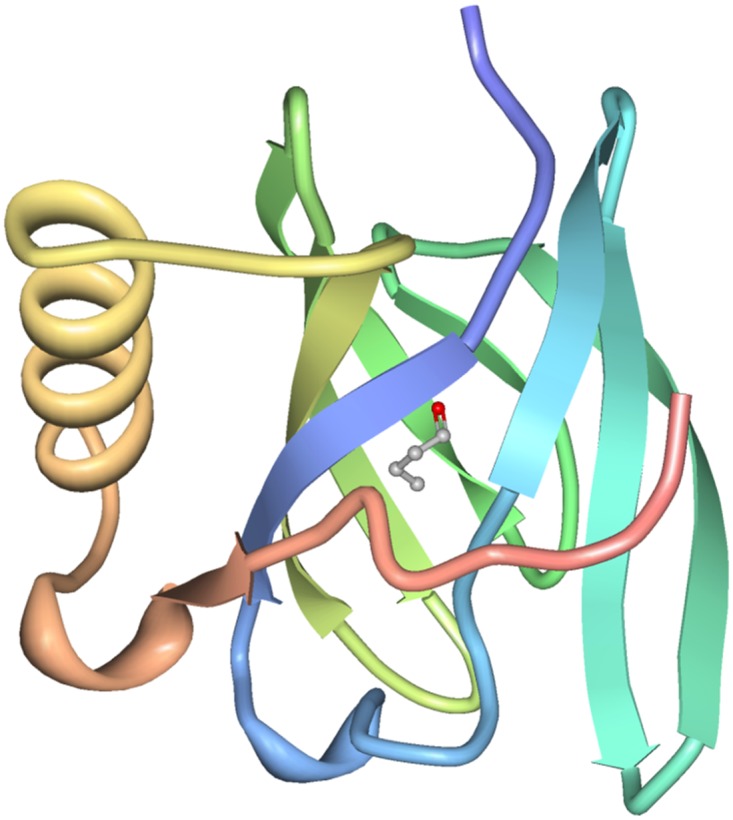
Porcine Odorant Binding Protein structure. Cartoon representation of the structure pOBP with butanal. The image was produced by Protein workshop Right.

The presence of molecules that compete with AMA molecules for the protein active site, i.e. with a higher affinity than AMA for the protein active centre, leads to the displacement of AMA molecules and the interruption of the energy transfer phenomenon (see Fig. 3). Before performing the competitive assay with butanal by using pOBP on the POF chip derivatized with butyric acid, we performed a functional assay on pOBP using the fluorescent ligand 1-amino-anthracene (AMA) and incubation with butanal in order to verify the affinity of the pOBP for the selected analytes. The achieved results demonstrate a good affinity of pOBP for butanal (K_D_ value of 1.92 μM).

### 3. Binding experiments

**Fig 4:**
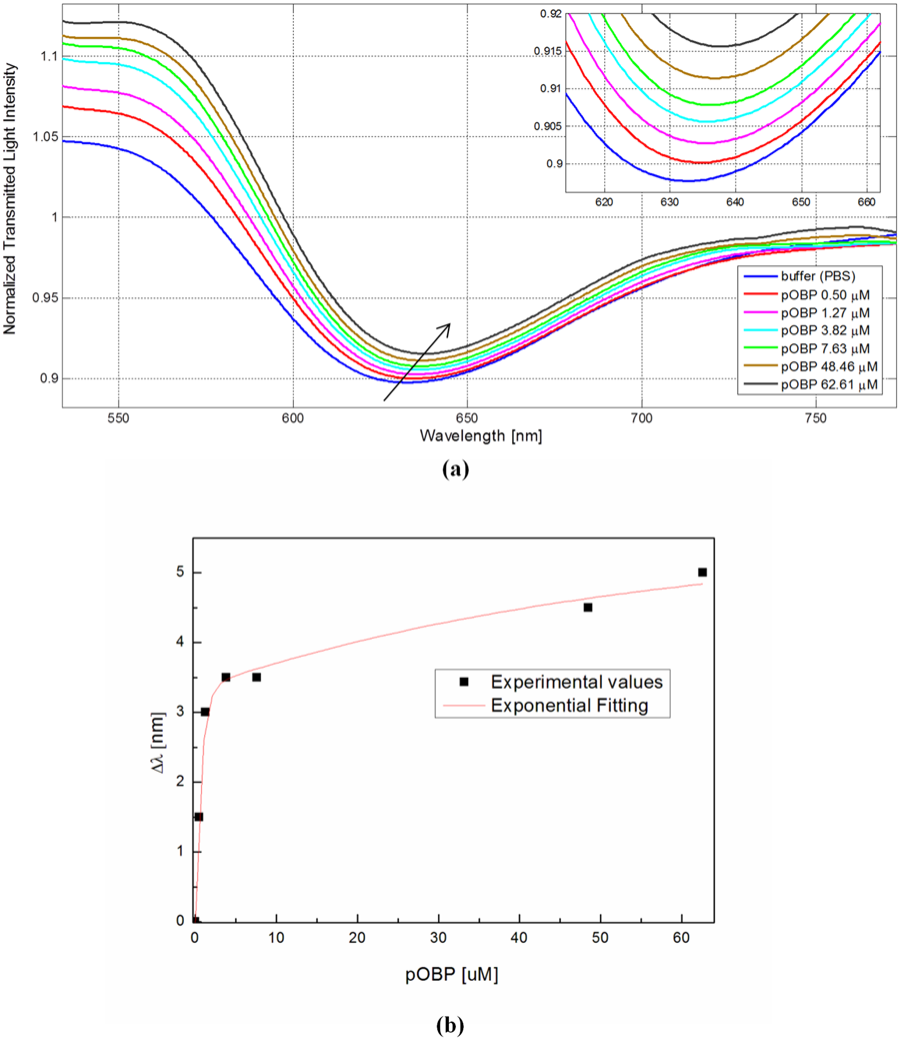
Binding experiment-SPR results. (a) SPR transmission spectra, normalized to the air spectrum, for different pOBP concentrations in the case of sensor with gold layer with bio-receptor. Inset: Zoom of the resonance wavelengths region. (b) Resonance wavelength variation as a function of pOBP concentrations [μM].

Fig. 4a shows the transmission spectra, normalized to the spectrum achieved with air as the surrounding medium, of the sensor in aqueous solution obtained by contacting standard solutions at increasing concentrations of pOBP. The resonance wavelength is shifted to higher values by increasing the concentration of pOBP, which demonstrates that pOBP is adsorbed at the derivatized sensor surface, clearly producing an increase of the refraction index of the medium. Fig. 4b shows the resonance wavelength variation, evaluated searching the dip of the spectrum, as for instance that reported in Fig. 4a, by Matlab software, versus the different concentrations of pOBP. In the same figure, the exponential fitting to the experimental data is presented too.

**Fig 5:**
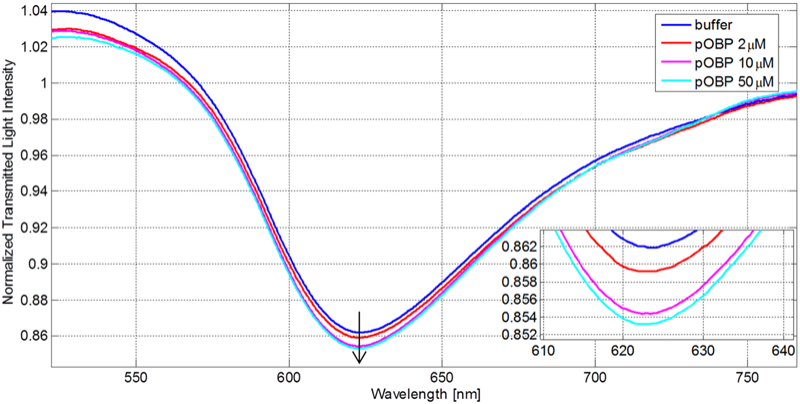
Control—SPR experimental results. SPR transmission spectra, normalized to the air spectrum, for different pOBP concentrations in the case of the reference sensor with gold layer—SAM without bio-receptor. Inset: Zoom of the resonance wavelengths region.

A control experiment was also carried out, in which the response of a sensor based on SPR in POF without the receptor (butyric acid) has been studied. The spectra recorded for the same solutions at different pOBP concentrations, are reported in Fig. 5 for comparison purposes. No wavelength shift at increasing concentrations of pOBP is observed for the reference sensor, indicating that the signal, *i.e*., the resonance wavelength variation, Δλ, is only produced by the interaction of pOBP with the receptor.

### 4. Competitive assay

**Fig 6:**
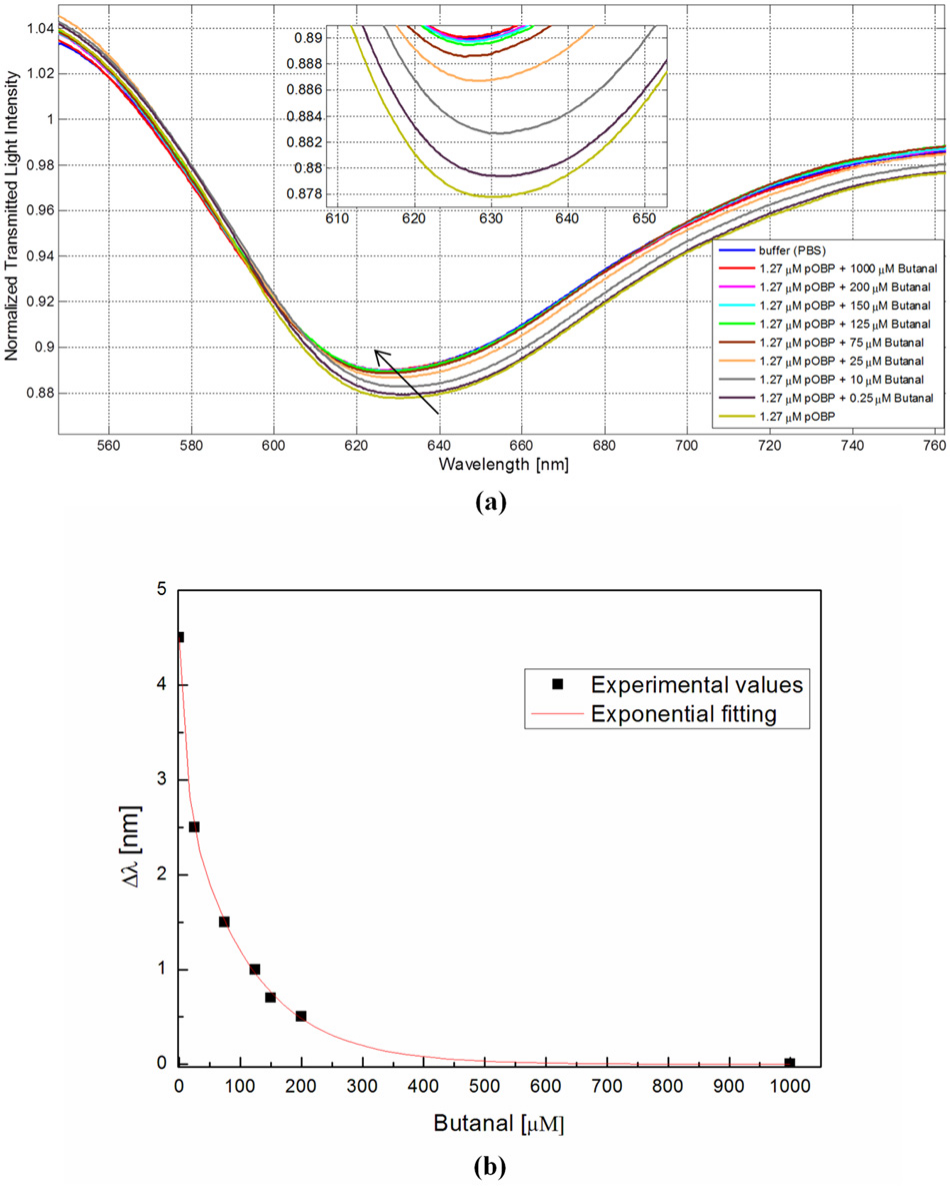
Competitive assay-SPR. (a) SPR transmission spectra, normalized to the air spectrum, for different concentration in solution of butanal and with a fixed concentrations of pOBP (1.27 μM), in the case of sensor with gold layer with bio-receptor. Inset: Zoom of the resonance wavelengths region. (b) Resonance wavelength variation as a function of butanal concentration, obtained with binding of a fixed concentration of pOBP in presence of increasing concentration of butanal during pre-incubation time.

Fig. 6a shows the transmission spectra, normalized to the reference spectrum (achieved with air as the surrounding medium), of the sensor when different concentrations of butanal are pre-incubated with a fixed concentration of pOBP (1.27 μM). The resonance wavelength is shifted to higher values by decreasing the concentration of butanal in the solution with pOBP. In Fig. 6b, the resonance wavelength variation is presented, versus different concentrations of butanal, obtained with different concentrations of butanal pre-incubated with pOBP (1.27 μM), together with the exponential fitting to the experimental data.

The experimental results show the selective detection of butanal using an optical platform with butyric acid-SAM layer on the gold surface and pOBP. The selectivity to one solely specific target is induced by a specific property of OBPs, which bind selectively one analyte and are not sensitive to molecules other than the one to which they are specifically tuned for selective bio/recognition. Such bio/receptors, by specifically tuning their binding properties, then allow achievement of highly selective detection that is insensitive to an interfering agent and unspecific adsorption as a result of their selectivity-induced recognition properties [17–19].

In the first step, the measurements have shown the binding between pOBP and the receptor (butyric acid-SAM layer) immobilized on the gold surface. The results are summarized in Fig. 4a and 4b. In this case, when the pOBP concentration increases, the resonance wavelength shifts toward higher values (clearly indicating an increase of the medium refractive index). Fig. 5 shows that this phenomenon is not present when on the gold layer the receptor (butyric acid) is not present. This last test is instrumental to assess the sensor’s chemical specificity.

In the second step, the measurements have been obtained with different concentrations of butanal pre-incubated with a fixed concentration of pOBP (1.27 μM). After the pre-incubation, the pOBP binds with butanal (analyte) and it is not available for binding with the receptor. In this case when in the solution with pOBP the butanal concentration decreases, the resonance wavelength shifts toward higher values, because the binding between pOBP and the receptor increases (see Fig. 6). Fig. 6 shows a large shift of the resonance wavelength in the presence of butanal at a concentration of 25μM. This concentration value has been used to define the poorest limit of detection. Other methods present a smaller detection limit of butanal (about one order of magnitude smaller), as reference 1 shows.

## Conclusions

This work presents a new optical biosensor based on the SPR-POF platform, the butanal and the pOBP. The sensing method of the biosensor is based on a competitive assay. The obtained results show that the POF-Butyric acid-pOBP biosensor is able to sense the butanal in aqueous solution in the range of concentrations between about 20 μM and 1000 μM. The proposed sensing methodology could be of easy implementation in different application fields.

The developed method points out a strategic approach to reduce the cost and the size of sensing by offering the possibility to integrate it into a “Lab-on-a-chip” platform.

## Experimental

### Materials

N-hydroxysuccinimide (NHS), N- (3-dimethylaminopropyl)-N’-ethylcarbodiimide hydrochloride (EDC), butyric acid, ethylenediamine and α-lipoic acid were purchased from Sigma-Aldrich (Sigma-Aldrich S.r.l., Milan, Italy). All other chemicals were commercial samples of the purest quality.

### Porcine Odorant Binding Protein purification

Expression of the porcine odorant-binding protein (pOBP) was done in *E. coli* BL21-DE3 cells transformed with the pGEX2TK expression vector containing the gene encoding the fusion protein. The cells were picked and inoculated in 2.5 mL LB medium (Lysogeny broth medium) with ampicillin at the final concentration of 50 μg/mL. The culture was incubated over night at 37°C under shaking at speed of 160 rpm. Then, the culture was inoculated in 0.5 L of fresh LB medium containing ampicillin at the final concentration of 50 μg/mL, and was incubated at 37°C under shaking at speed of 160 rpm. When the culture absorbance value at 600 nm was 1.0 O.D, the expression of the gene encoding recombinant protein was induced with isopropyl-β-D-1-thiogalactopyranoside (IPTG) at the final concentration of 0.5 mM. After 3h of the post-induction incubation under shaking, the bacterial suspension was centrifuged at 3500 rpm for 30 minutes at 4°C, and the cellular pellet obtained was re-suspended in Lysis Buffer (PBS at pH 7.3) in a ratio: 1g pellet/3mL Lysis Buffer. Finally Lysozyme 0.4%, and, then DNAsi I (0.05mg/mL) and MgCl_2_ (5mM), were added and the two incubation intervals of 30 minutes either at 37°C under shaking were needed. The cells were destroyed by French Press and were centrifuged at 30000 rpm for 30 minutes at 4°C. Before the purification step, the sample was concentrated by ultrafiltration using Millipore’s Ultracel YM cellulose membrane with a cut-off of 30000 NMWL. The purification of the protein was performed by a GST-Affinity Chromatography using a Glutathione Sepharose 4 Fast Flow (GE Healthcare, Life Sciences at Biocompare.com.) according to the manufacturer’s instructions. Briefly the protein sample was loaded on the column of Glutathione Sepharose 4 Fast Flow and incubated at room temperature for 30 minutes under shaking. After this incubation step, the column was washed with the Binding Buffer (PBS at pH 7.4) in order to remove protein contaminants that are not bound to the resin. This binding step is follow with incubated of the column with thrombin (1 unit of thrombin size 100 μg of fusion protein) for 16 hours at 25°C. After 16 hours the column wash with a Binding Buffer (PBS 1X pH 7,3) and collect the protein. The column was regenerated by elution of the GST fusion protein with an Elution Buffer (50 mM Tris-HCl, 10 mM reduced glutathione pH 8.0). The elution fractions of pOBP were monitored by absorbance at λ 278 nm and SDS PAGE (12% Acrilamide) was carried out to evaluate the purity of the samples (data not shown). The obtained samples were collected and dialyzed against PBS at pH 7.3.

### Binding of pOBP to butanal

The capability of the pOBP to bind and recognize butanal was verified by 1-AMA binding assay. Briefly, 1.0 ml of 1.0 μM pOBP, in 20 mM Tris–HCl buffer pH 7.8, was incubated overnight at 4°C in the presence of increasing concentrations of AMA (0.156–10 mM). Fluorescence emission spectra were recorded between 450 nm and 550 nm by fluorometer (ISS K2 model) (excitation and emission slits were set at 2.0 nm) at a fixed excitation wavelength of 380 nm. The formation of the AMA-OBPs complex was followed by the increase of the fluorescence emission intensity at 480 nm.

### Optical Platform and experimental setup

The fabricated optical sensor platform was realized by removing the cladding of a plastic optical fiber along half the circumference, spin coating on the exposed core a buffer of Microposit S1813 photoresist, and finally sputtering a thin gold film using a sputtering machine [20]. The planar gold layer can be employed for depositing a receptor layer. In this case the selectivity detection of analyte is possible.

The plastic optical fiber has a PMMA core of 980 μm and a fluorinated polymer cladding of 20 μm. The thickness of the photoresist buffer was about 1.5 μm. The gold film so obtained was 60 nm thick. The realized sensing region was about 10 mm in length. The refractive indexes of the materials, in the visible range of interest, are about 1.49 for PMMA, 1.41 for fluorinated polymer and 1.61 for Microposit S1813 photoresist.

The experimental setup was arranged to measure the transmitted light spectrum and was characterized by a halogen lamp, illuminating the optical biosensor systems (POF of 1,000 μm in diameter), and a spectrum analyzer, as shown in Fig. 1.

The employed halogen lamp (Model no. HL-2000-LL, manufactured by Ocean Optics, Dunedin, FL, USA) exhibits a wavelength emission range from 360 nm to 1,700 nm, while the spectrum analyzer detection range was from 200 nm to 850 nm. An Ocean Optics “USB2000+UV-VIS” spectrometer was employed. The spectrometer was finally connected to a computer.

The SPR curves along with data values were displayed online on the computer screen and saved with the help of advanced software provided by Ocean Optics. The SPR transmission spectra, normalized to the spectrum achieved with air as the surrounding medium, are obtained using the Matlab software and the resonance wavelength was extracted for the analytical information.

### Immobilization process of butyric acid on the chip surface

The surface of the POF chip was sequentially cleaned with: (1) milli-Q water (3 times for 5 minutes) and (2) 10% of ethanol solution in milli-Q water (3 times for 5 min).

The surface of the chip was pre-treated before the covalently immobilization of butyric acid and the procedure consists of three different steps: (1) thiol film production, (2) derivatization of the surface with ethylenediamine and (3) butyric acid immobilization by EDC/NHS methods.

In the first step the gold chip was immersed in freshly prepared solution of α-lipoic acid dissolved in a solution of pure ethanol 10% in water at the final concentration of 40 mM and incubated at room temperature for 18 h. After this period of incubation, the gold-coated substrates surface was washed with milli-Q water and incubated over-night at room temperature with a mixture of EDC/NHS at the final concentration of 20 mM and 50 mM respectively dissolved in potassium phosphate buffer 100 mM at pH 5.5. After this step the surface was incubated with a solution of ethylenediamine 20 mM for 4 hours at room temperature. The surface was washed extensively in order to remove the excess of ethylenediamine.

The final step was the incubation overnight of the surface with a solution 50 mM of butyric acid, pre-incubated for 1h at room temperature with a mixture of EDC/NHS (20 mM and 50 mM respectively) in Potassium Phosphate buffer 100 mM at pH 5.5, at room temperature. The chips were at the end of these treatment washed with potassium phosphate buffer 100 mM at pH 5.5 (three times), with milli-Q water and finally dried with nitrogen.

### Binding experiments

Surface plasmon resonance experiments were carried out on SPR-POF chip; each chip was covered with a thin film of parafilm to draw a lane on the fiber to allow the deposition of all sample with a final volume of 50 μL. Measurements were performed in phosphate buffer saline pH 7.4 (PBS) at room temperature. The derivatizated surface of the chip was incubated with increased concentration of pOBP in the range from 0 to 62 μM. The sample was incubated 15 minutes and SPR measurements were carried out. After the measurements the chip was extensively washed with PBS buffer.

### Competitive assay

A competition binding SPR assay was carried out at fixed pOBP concentration of 1.27 μM in absence or in the presence of an increasing concentration of butanal (butyraldehyde). The range of concentration was from 0.25 μM up to 1000 μM diluted in milli-Q water. The incubation was done at room temperature for 30 minutes in eppendorf tube. After this pre-incubation in presence of butanal, all samples of pOBP/butanal were incubated with butyric acid-derivatized surface of chip POF and SPR measurements were carried out after 15 minute of incubation time.

### AFM characterization

The surface sample morphology was investigated by atomic force microscopy (AFM) using a Digital Instruments D3100 AFM equipped with a Nanoscope IIIa controller. The AFM was operated in air in Tapping Mode at a resonance frequency of about 250 kHz employing commercial silicon probes with apex curvature radius of ~5–10nm. AFM scans with size in the range between 500nm and 10 μm were collected. The sample roughness was estimated by evaluating the root mean square (RMS) roughness (Rq), i.e. the root mean square average of the topography height coordinates.
